# Successful removal of an impacted metallic arrowhead penetrating up to the brainstem

**DOI:** 10.4103/0974-2700.66566

**Published:** 2010

**Authors:** Dharmdas Paramhans, Sapna Shukla, Ankur Batra, Raj K Mathur

**Affiliations:** Department of Surgery, M.G.M. Medical College, Indore, MP, India

**Keywords:** Arrow injuries, arrowhead traversing brainstem, base of skull

## Abstract

A case of impacted metallic arrowhead in the brain through an unusual route of the neck and behind the external carotid artery to the base of the skull up to the brainstem is reported. Review of the literature reveals no previous reports of this type of injury. A 35-year-old man was admitted to the hospital after 36 h of injury, being fully conscious and with partial facial palsy. The arrowhead was successfully removed by exploration of the entry wound, without any neurovascular complications. The patient not only survived the operation but was also discharged in an improved neurological condition.

## INTRODUCTION

Although arrow injuries are a rarity in the west, they are still seen among the tribal population of India. Most of them are over the chest and abdomen; those over the head and neck are uncommon. Penetrating neck injuries remain challenging as there are a number of important structures in a small area and injury to any of these structures may not be readily apparent. Penetrating impalement wounds usually occur secondary to a fall onto a piercing object or are sustained from machinery or pneumatic tools, but also include low-velocity missiles such as arrows. The offending agent is plunged into the body along the long axis of the blade, resulting in a small puncture wound of the skin and unknown depth. Of these wounds, 4% mortality is primarily from direct injury to the great vessels. The arrowhead piercing through the neck and base of the skull may damage neurovascular structures lethal to life. During retrieval, fierce hemorrhage from major vessels can occur. Manipulation inside the brain itself may result in neurological deficiency.

## CASE REPORT

A 35-year-old male from a tribal area sustained an arrow injury in the left side of his upper neck, penetrating through the base of the skull and into the brain. He was unable to move his neck and had severe pain. He reached our center nearly 36 h after initial resuscitation.

On examination, he was hemodynamically stable, had partial facial palsy and was unable to open his jaw. The entry wound was behind the left angle of the mandible, with only 1 cm of the arrowhead visible from the outside. Neurologically, he was fully conscious and alert, with Glassgow Coma Scale (GCS) 15/15. He had no diplopia or voluntary down gaze. He could walk with support but did not have weakness in the legs and hips. There was no spasticity, rigidity, involuntary movements, dystonia, resting tremors or myoclonus. Finger to nose and heel to shin examinations were normal. No hyper/hyporeflexia or Babinski sign was found.

**Figure 1:**
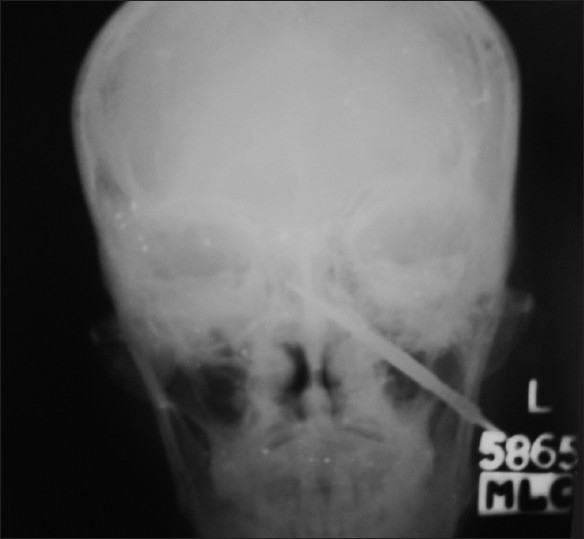
X-ray of the skull showing a 10-cm-long arrowhead penetrating the base of the skull from below the mastoid process obliquely and reaching inside the middle cranial fossa

**Figure 2:**
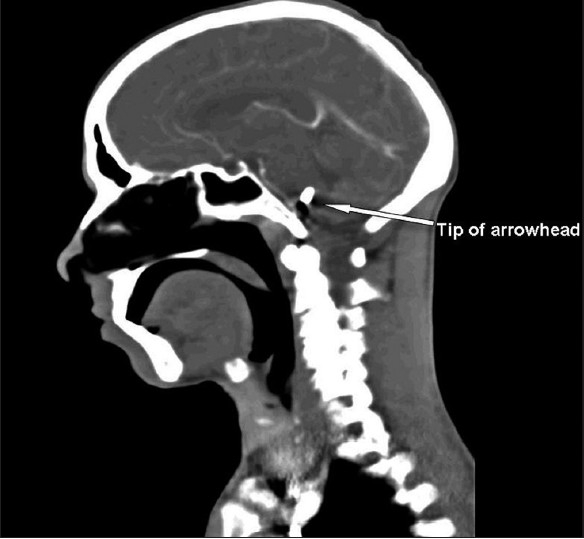
Computed tomography of the brain showing the tip of the arrowhead reaching up to the brainstem

X-ray of the skull showed a 10-cm-long arrowhead penetrating the base of the skull from below the mastoid process to the middle cranial fossa [Figure 1]. Computed tomography (CT) of the brain showed the tip of the arrowhead reaching up to the brainstem [Figure 2], and his color Doppler was normal.

**Figure 3:**
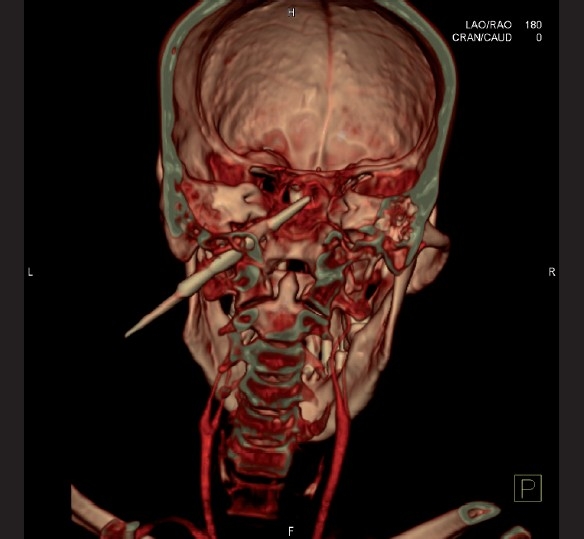
3D-computed tomography images showing the track of the arrowhead traversing just above the bifurcation of the common carotid

3D-CT images showed the track of the arrowhead traversing just above the bifurcation of the common carotid [Figure 3].

In our case, nearly 8 cm of the arrowhead was inside the point of penetration and about 4 cm of it penetrated into the base of the brain.

### Surgical approach

**Figure 4:**
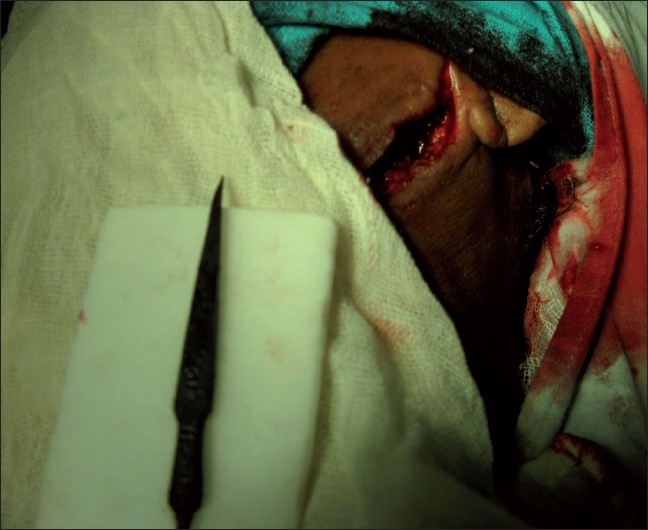
Arrowhead after retrieval

Positioned supine on the operating table with extension of the neck and rotation to the contralateral side, an oblique vertical incision along the anterior border of the sternocleidomastoid was given. The carotid vessels and Internal Jugular Vein (IJV) were isolated and safeguarded. Because of the major part being stuck in, it was gently dislodged and retrieved [Figure 4].

The post-operative recovery was uneventful, without any neurovascular deficit, and the patient gradually recovered from facial palsy. He was followed-up for 6 months.

## DISCUSSION

Arrow wounds are very rare in western countries, but they are still prevalent in the Indian tribal population.[1] Most commonly, arrow injuries are seen over the chest and abdomen, but are less common in the head and neck region. While retained foreign bodies into the brain, like bullets, pellets and glass pieces are not unusual in themselves, there is little data on the incidence of projectile, penetrating non-gunshot head trauma.[2,3] The route that this foreign body has traversed makes it an interesting case study.

### Mechanism of injury

Arrows have a considerable penetrating capacity in soft tissueand flat bones, sufficient to penetrate deeply into large bodycavities. Injuries caused by arrows are usually less destructive thanthose caused by bullets because of the lower velocity and energy. Barbed arrows are an exception because of the risk of extensivedamage to major structures when retrieved.[5] They can generate velocities up to 74 m/s (240 ft/s). These produce impalement wounds in which the offending agent is plunged into the victim along the axis of the blade, resulting in a small puncture wound of the skin and unknown depth. An impaling object can provide tamponade of major vessels and, therefore, should be removed under direct vision.[4]

The neck has a high density of vital structures that, when injured, can threaten both life and function. Although major vascular injuries may be readily recognized on presentation, aerodigestive and neurologic injuries may be more difficult to detect. As in the management of any injury, airway compromise is the primary concern and may be particularly challenging in the patient with a penetrating neck injury.

These patients should be resuscitated according to the Advanced Trauma Life Support (ATLS) protocol.[7] The primary survey, including airway management with cervical spine immobilization, breathing and ventilation and circulation restoration, is carried out to stabilize the patient. Disability of the nervous system is assessed by the Alert, Voice, Pain, Unresponsive scale (AVPU) score and GCS. X-rays, CT scans and focused abdominal sonography for trauma are performed to evaluate internal injuries. The secondary survey is carried out for anatomical and physiological evaluation of the head and neck for fractures and responses, of the chest (front and back) for respiratory distress or decreased air entry, of the abdomen and pelvis for visceral injuries and of the limbs for any deformities or neurovascular deficiency.

In evaluating penetrating injuries of the neck, history of loss of consciousness, significant blood loss, hemoptysis, hematemesis and voice change is important. In analyzing wounds based on craniocaudal location, the neck is divided into three horizontal zones: Zone I, from sternal notch to cricoid cartilage; Zone II, from cricoid to angle of mandible; Zone III, from angle of mandible to base of skull. Penetrating wounds may traverse more than one zone and require evaluation of all possible structures in all affected zones.[10]

Patients with Zone I and Zone III injuries should undergo pre-operative imaging to allow appropriate operative planning to assess the need for proximal and distal control via incisions other than anterior neck incision.[8] Four-vessel angiography is considered useful as a roadmap prior to surgery. But, it had a low yield, with potential morbidity, which led surgeons to search for other options for vascular evaluation. The non-invasive color flow Doppler and CT-angiography have proved very useful.[9]

### Operative exposure

The patient is generally positioned supine with extension of the neck and rotation to the contralateral side if cervical spine injury is cleared. Active bleeding should be controlled with digital pressure until direct vascular control is achieved. Most penetrating neck injuries are initially approached via an incision along the anterior border of the sternocleidomastoid muscle. The most difficult exposure is Zone III injury, in which distal control of the vasculature is impeded by the base of the skull. Arterial injuries can be managed by primary closure in cases without tension. Patch angioplasty and segmental resection with primary anastomosis are also viable options in the stable patient. Bleeding from the internal carotid artery at the base of the skull can be temporarily controlled with balloon occlusion. Unilateral ligation of the vertebral artery rarely results in a neurologic deficit; otherwise, arterial embolization should be attempted. For patients in shock, venous injury should be managed with ligation. Neck wounds showing increased bleeding after positive-pressure ventilation indicate venous injuries. Internal jugular venous injury is managed by lateral venorrhaphy and post-operative systemic anticoagulation. External jugular venous injury can always be managed with ligation.

An arrow should never be removed from a patientwith stable or unstable vital signs before an injury to themajor blood vessels has been ruled out.[4] Penetrating injuries to the brain by high-velocity arrows may require some management strategies that differ from those due to injuries caused by gunshot wounds.[6] In our case, the retrieval of impacted arrow by craniotomy could not have been feasible as the broad end was outside the skull base and was close to the carotid vessels. To reach the brainstem itself and its manipulation to hold the pointed end of the foreign body would have resulted in disastrous results. The lower facial muscle weakness in this patient may be due to trivial injury to the corticobulbar fibers originating from the brainstem above the level of the facial nucleus. Muscles of the head, except for the lower facial muscles, receive both crossed and uncrossed corticobulbar fibers.[11] In this case, a follow-up period of 6 months was uneventful.

## CONCLUSION

Penetrating neck injuries are life-threatening due to the density of vital structures in the narrow space. They produce a tamponade effect after impalement, and retrieval should be performed only after pre-operative imaging of neurovascular structures. Initial resuscitator management according to the ATLS system helps in stabilizing the patient and improving outcome. Pulsatile, expanding hematoma and increasing subcutaneous emphysema needs immediate exploration in the operation theater. Zone I and Zone III neck region explorations need structure identification and may require separate incisions.
